# National Variation in EMS Response and Antiepileptic Medication Administration for Children with Seizures in the Prehospital Setting

**DOI:** 10.5811/westjem.59396

**Published:** 2023-07-17

**Authors:** Maytal T. Firnberg, E. Brooke Lerner, Nan Nan, Chang-Xing Ma, Manish I. Shah, N. Clay Mann, Peter S. Dayan

**Affiliations:** * Columbia University Irving Medical Center, New York, NY; † State University of New York at Buffalo, Buffalo, NY; ‡ Baylor College of Medicine and Texas Children’s Hospital, Houston, Texas; § University of Utah, Salt Lake City, Utah

## Abstract

**Background and Objectives:**

Prehospital Advanced Life Support (ALS) is important to improve patient outcomes in children with seizures, yet data is limited regarding national prehospital variation in ALS response for these children. We aimed to determine the variation in ALS response and prehospital administration of antiepileptic medication for children with seizures across the United States.

**Methods:**

We analyzed children <19 years with 9-1-1 dispatch codes for seizure in the 2019 National Emergency Medical Services Information System dataset. We defined ALS response as ALS-paramedic, ALS-Advanced Emergency Medical Technician, or ALS-intermediate responses. We conducted regression analyses to identify associations between ALS response (primary outcome), antiepileptic administration (secondary outcome) and age, gender, location, and US census regions.

**Results:**

Of 147,821 pediatric calls for seizures, 88% received ALS responses. Receipt of ALS response was associated with urbanicity, with wilderness (adjusted odds ratio [aOR] 0.44, 0.39–0.49) and rural (aOR 0.80, 0.75–0.84) locations less likely to have ALS responses than urban areas. Of 129,733 emergency medical service (EMS) activations with an ALS responder’s impression of seizure, antiepileptic medications were administered in 9%. Medication administration was independently associated with age (aOR 1.008, 95% confidence interval [CI] 1.005–1.010) and gender (aOR 1.22, 95% CI 1.18–1.27), with females receiving medications more than males. Of the 11,698 children who received antiepileptic medications, midazolam was the most commonly used (83%).

**Conclusion:**

The majority of children in the US receive ALS responses for seizures. Although medications are infrequently administered, the majority who received medications had midazolam given, which is the current standard of care. Further research should determine the proportion of children who are continuing to seize upon EMS arrival and would most benefit from immediate treatment.

## INTRODUCTION

Emergency medical services (EMS) personnel transport approximately three million children annually in the United States, representing 10% of all EMS transports.^1^ Seizures account for 10% of these pediatric EMS encounters.^2^ While many seizures resolve spontaneously and quickly without intervention, rapid advanced-level care can be critical for improving outcomes in those who do not. The likelihood of seizure cessation, either spontaneously or with antiepileptic treatment, decreases with longer seizure duration.^3^,^4^ Prolonged seizure duration is associated with both increased morbidity, such as chronic neurological deficit, and mortality.^5^

Seizure duration is one potentially modifiable factor that can affect outcomes, and most current treatment protocols recommend that the first antiepileptic medication be administered within five minutes of seizure onset with a second medication given by 10 minutes.^6^ Seizure treatment requires Advanced Life Support (ALS) training and skill, due to the potential necessity for administering controlled antiepileptic medications or the need for airway or circulatory system support, neither of which can be provided by a Basic Life Support (BLS) response. The EMS personnel certified in BLS do not carry antiepileptic medications. A prior study evaluating prehospital treatment of status epilepticus showed that timely ALS prehospital treatment improved patient outcomes.^7^

Studies regarding the prehospital management of children with seizures have been primarily at the patient level and describe local or regional care. Those studies have not reported national level data. 8–10 There is no published national data that describes the variation in prehospital level of care and management used for children with seizures. Nationwide, BLS personnel outnumber ALS and Intermediate Life Support personnel combined by a factor of approximately three to one.^11^ The distribution of these EMS personnel may introduce variability in EMS response type and management.

The National EMS Information System (NEMSIS) provides an opportunity to understand the variation in response across the country for children with high acuity complaints.^12^ Identifying the variation in EMS response and understanding current practices in prehospital management are important at both the patient and system level. At the patient level, the data may identify potential areas where the care is suboptimal, such as receiving medications that are not the standard of care or prolonged time to administration of medications. At the system level, this data may reveal opportunities for system- and regional–level improvements in protocols and policies. In this study, we aimed to determine the variation in types of EMS response to 9-1-1 calls for pediatric seizures across the US, based on patient demographics and geographic location. Secondarily, we aimed to determine the frequency with which children received an antiepileptic medication and whether this differed based on patient demographics and location.

**Population Health Research Capsule**

What do we already know about this issue?

*Timely pediatric seizure treatment is crucial to prevent morbidity and mortality. Variation in EMS seizure care underscores the need for systems improvement*.

What was the research question?

*Is there variation in EMS response and medication administration for pediatric seizures in the United States?*

What was the major finding of the study?

*Advanced Life Support response was 88%: lower in rural areas compared to urban (aOR 0.80). Antiepileptics were administered in 9%, slightly higher in older patients*.

How does this improve population health?

*This data enhances our understanding of national EMS responses and management for children with seizures based on patient geography and demographics*.

## METHODS

### Study Design

We conducted a retrospective, cross-sectional analysis of the NEMSIS public research dataset.^12^ Our institutional review board granted this study exempt status.

### Data Source

The NEMSIS project is a national effort to standardize EMS data collected by EMS personnel. The database for the project is managed by the NEMSIS Technical Assistance Center (currently located at the University of Utah School of Medicine, Salt Lake City, UT) and supported by the Office of Emergency Medical Services of the National Highway Traffic Safety Administration. Local EMS agencies submit data to their state database, of which a subset is then exported to the national database.^13^ For this study, we used the 2019 NEMSIS Public Release Dataset (version 3), which includes 22,532,890 EMS activations submitted by 9,599 EMS agencies serving 43 US states and territories during the 2019 calendar year.

### Participants: Selection of EMS Responses

We included children <19 years in age who accessed 9-1-1 EMS for complaints of seizures or convulsions during the 2019 calendar year. We excluded interfacility transfers. To identify relevant cases of EMS activations for the care of pediatric seizures, we used codes from the NEMSIS dataset that related to reason for dispatch as convulsion or seizures (eDispatch.01 - 2301025). To assess the potential differences between dispatch reason and the EMS responder’s impression (eSituation.11 and eSituation.12), we also identified children for whom the primary or secondary responder impression was documented as seizure or convulsion. These impressions were documented as International Classification of Diseases, 10^th^ Rev, Clinical Modification (ICD-10-CM) codes that were recorded by EMS personnel. We included all ICD-10-CM codes that refer to seizure, which include ICD-10-CM codes with the prefixes of R56, G40, and P90.

### Outcomes

The primary outcome of our study was receipt of ALS response for patients with dispatch complaints of seizures, as BLS personnel are unable to administer antiepileptic medications. We defined ALS response as any of the following: ALS-paramedic, ALS–Advanced Emergency Medical Technician (AEMT), or ALS-intermediate (as described within NEMSIS element eResponse.15). We defined BLS response as any of the following: BLS–Basic/ EMT, BLS-AEMT, BLS-First Responder, BLS–Intermediate (as described within eResponse.15).

The secondary outcome was receipt of an EMS-administered antiepileptic medication among patients with an ALS response and the EMS responder’s impression of seizure. We included all formulations of the following seizure abortive medications as documented in eMedication.03: midazolam, lorazepam, diazepam, levetiracetam, phenytoin, phenobarbital, and clonazepam. We assessed the timing of medication administration in two ways: a) calculated as the time from the time of dispatch (eTimes.03) to the time of medication administration (eMedication.01); and b) calculated as the time from EMS arrival on scene (eTimes.06) to the time of medication administration (eMedication.01). We calculated these two times as the former includes the time to arrive on the scene, while the second excludes this period.

### Covariates and Definitions

From the NEMSIS database, we collected the following covariates: patient demographics (age, gender, race, and ethnicity); insurance status (private insurance, Medicaid, self-pay, no insurance identified, other, or missing); census region (Midwest, Northeast, South, and West); and urbanicity based on the U.S Department of Agriculture Urban Influence Codes (urban, suburban, rural, and wilderness).

### Statistical Analysis

We summarized our data using standard descriptive statistics. For our primary outcome (receipt of ALS response), we conducted a multivariable logistic regression analysis to determine its relationship to the following covariates: patient demographics (age, gender); census region; and urbanicity. We excluded any variables that were missing >20%.

For our secondary outcome (receipt of antiepileptic medication), we analyzed only those patients for whom ALS care was dispatched and had an EMS responder’s impression of seizure. We conducted a logistic regression analysis to determine the relationship between receipt of an antiepileptic medication and age, gender, census region, and urbanicity. For all regression modeling, we assessed for multicollinearity using variance inflation factors and determined model goodness-of-fit. For variables that were collinear, we chose only one of the variables to further analyze.

Lastly, we calculated the number with EMS responder impression of seizure (eSituation.11 and .12) divided by number with EMS dispatch for seizure (eDispatch.01) to understand how frequently these impressions matched.

## RESULTS

**Figure 1:**
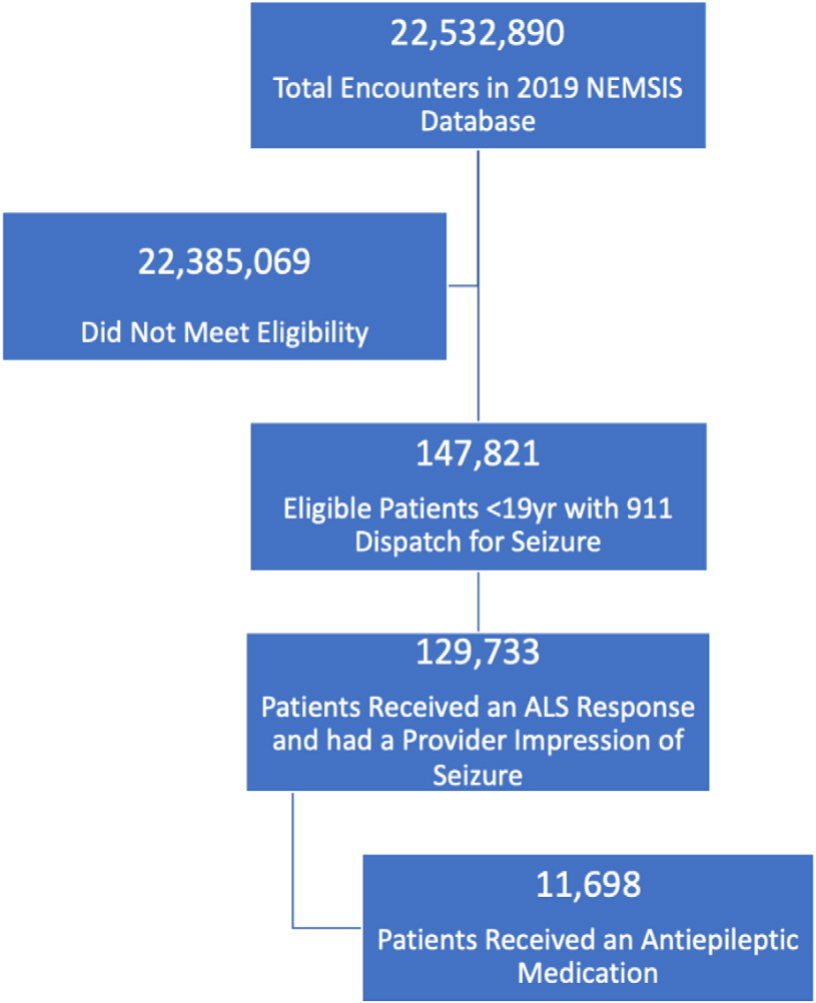
Patient flow diagram. *NEMSIS*, National Emergency Medical Services Information System; *ALS*, Advanced Life Support.

We identified 147,821 eligible EMS activations for pediatric patients <19 years of age with 9-1-1 dispatch for seizure or convulsion. Figure 1 and Table 1 note that the majority of children received an ALS response (88%), which was evenly distributed by age and gender. Most encounters overall occurred in urban areas (84%). Most patients had missing or unknown data for race, ethnicity, and insurance status; therefore, this data was not analyzed further. In the multivariable analysis (Table 2), receipt of ALS response increased marginally for every year increase in age and decreased for those living in rural or wilderness locations.

We identified 11,698 EMS activations for which patients received an antiepileptic medication, representing 9% of encounters that received an ALS response and had an EMS responder’s impression of seizure. The administration of an antiepileptic medication was evenly distributed across all age groups, regions, and urbanicity (Table 3). In the multivariable analysis (Table 4), receipt of an antiepileptic medication was significantly higher in females compared to males.

Of the 147,821 EMS activations that had a dispatch indication of seizure, 109,684 (74%) also had an EMS responder’s primary or secondary impression of seizure. Midazolam was the most commonly administered medication, comprising 83% of all antiepileptic medications administered (Supplement 2). The median documented time from dispatch to medication administration for all encounters was 16.75 minutes (Table 5). Urban areas had the fastest times to medication administration (median 16.44 minutes [min]) and wilderness the slowest (median 21.00 min). The median time to administration of antiepileptic medication from dispatch was most rapid for children in the 1–4 years age group (median 15.82 min) and slowest in those 15–18 years of age (median 18.00 min).

The median documented time from EMS arrival on scene to medication administration was 9.63 min (Table 6). From EMS arrival on scene to medication administration, children in the 1–4 years age group had the fastest medication administration time (median 8.68 min), while children in the 10–14 years age group had the slowest (median 10.53 min). Upon regression analysis, (Table 7), time to medication administration was significantly faster in urban areas and slower as patient age increased.

## DISCUSSION

In this retrospective analysis of the NEMSIS database, most children with a dispatch code for seizure received an ALS response. Although ALS responses were statistically less frequent in rural and wilderness settings, it is notable that even in wilderness settings, more than three-quarters of EMS activations for children received ALS responses. This is reassuring as BLS personnel are unable to administer antiepileptic medications, and at the time of an EMS call for seizure it is unknown whether the seizure will self-resolve, continue, or recur. As expected, seizure abortive medications were administered to a relatively small fraction (~9%) of children receiving an ALS response for seizures, consistent with previously reported rates of active pediatric seizures in the prehospital setting.^14^ Despite the relatively small proportion of seizures that receive medications, those that do not self-resolve quickly may progress to result in significant morbidity and mortality without intervention.^3^–^6^

Midazolam was the most commonly administered medication across all regions and urbanicities, possibly due to the ability to deliver it by many routes and its heat-stability relative to other benzodiazepines.^15^ While there was modest variation in median times from dispatch to medication administration based on age, region, and urbanicity, median times across locations were beyond typical seizure durations for which abortive antiepileptic medications are considered optimal.^5^ The times to medication administration were not unexpected given that they include response time to the scene, time to access the patient, and initial evaluation. Similarly, there was modest variation in median times from EMS arrival on scene to medication administration based on age, region, and urbanicity. The median time of 9.63 min for all patients may be an opportunity for further research into causes of possible medication administration delays.

Prior data on ALS response as well as the receipt and timing of seizure medication administration in the prehospital setting has been limited due to the lack of national data-aggregation efforts. However, smaller scale studies noted a similar median time to intervention. This includes one study of three urban EMS systems that reported a 14-min median time to intervention after arrival on scene.^14^ Another study of nine children’s hospitals found a 30-minute median time from onset of seizures to administration of antiepileptic drugs in the outpatient setting.^8^ Our data provides a larger perspective of seizure management nationally and reflects both the time to reach a patient after dispatch and the time EMS arrives on scene to medication administration. Given that the time to medication administration exceeds the optimal time at which medication is expected to have effect, our data strongly suggests the need for abortive medications to be readily available in the homes of children with seizure disorders, including newer products such as intranasal midazolam and diazepam. Prior studies have demonstrated reduction in morbidity and costs associated with hospital visits when seizure abortive medication is available for at-home administration.^16^

The high frequency of midazolam use across patient demographics and geography suggests adoption of best clinical practice and recommendations.^9^ Among benzodiazepines, midazolam can be administered by multiple routes, including intramuscular and intranasal, which bypasses the potentially time-intensive step of obtaining peripheral intravenous (IV) access. In 2012, publication of the results of the Rapid Anticonvulsant Medication Prior to Arrival Trial (RAMPART), a large, multicenter, randomized clinical trial of prehospital patients with seizures, demonstrated that intramuscular midazolam was superior to IV lorazepam at terminating convulsions prior to arrival to the emergency department.^17^,^18^ A national study using NEMSIS demonstrated substantial increase in midazolam use after the publication of RAMPART.^19^ Another study of three EMS agencies in an urban setting similarly found high rates of midazolam use.^14^ Our data did note that urban settings had the highest proportionate use of midazolam, which could reflect the contributions of medical directors with board-certification in EMS at urban academic institutions or more practical issues related to availability or storage.

The development and growth of NEMSIS allows for a robust assessment of national prehospital data. In this study, NEMSIS contributed to our understanding of national EMS responses for higher acuity pediatric complaints based on patient geography and demographics. The American Academy of Pediatrics and other professional organizations have recommended that EMS systems and agencies contribute to the database.^20^ As NEMSIS expands and the data available becomes more granular, other studies regarding resource use and the quality of care will become increasingly possible to better understand trends and opportunities for improvement in pediatric prehospital care. For example, we were unable to analyze insurance status or race and ethnicity due to large amounts of missing data. As EMS documentation advances and NEMSIS becomes more complete, future research will be able to determine equity of access to high level pediatric care.

## LIMITATIONS

Our study has limitations inherent to retrospective large database studies. Identification of the target population can be challenging, as the accuracy of documentation collected and catalogued within NEMSIS could not be verified. However, by capturing both reason for dispatch and EMS responder impression, we were able to best estimate the degree to which our target population may have represented those who had seizures. Although it was impossible to identify those who actually seized during EMS care or the etiology of their seizure, our data is consistent with smaller research studies on this topic. Regardless of etiology of the seizure, prior data suggests that seizures of longer duration are harder to abort and are associated with higher morbidity and mortality with recommendations to administer abortive medications within five minutes.

The lack of standardization across EMS electronic health record systems and documentation affects data consistency. We attempted to overcome the potential variation in identifying children with seizures by using an inclusive list of ICD-10-CM codes. However, different agencies may have used different codes when mapping their locally collected data into the EMS database. Additionally, we recognize that the database did not allow us to ascertain whether children who received an antiepileptic medication required one and whether children who did not receive an antiepileptic medication did not require one. However, the similar proportion of children receiving abortive therapy across geographic locations is reassuring.

Finally, an important potential limitation is that ALS and BLS may be defined differently across EMS agencies and geography such that truncating multiple categories of EMS professionals into ALS and BLS may not reflect the differences in capabilities across responders (e.g. ability to provide medications) across settings. Further, from our data, we were unable to differentiate between patients served in a two-tiered EMS system and those who were not.

## CONCLUSION

The majority of pediatric patients across the US received an Advanced Life Support response for seizures; however, those in rural or wilderness settings were somewhat less likely to receive an ALS response. Although medications are infrequently administered, the majority of those who were administered a medication received midazolam, which is the current standard of care. Further research is required to determine the proportion of children who are continuing to seize upon EMS arrival and would most benefit from immediate and appropriate treatment.

## Supplementary Information



## Figures and Tables

**Table 1:** Level of emergency medical services care for children with dispatch seizure code.

	All Patients N = 147,821	Response Unit Level of Care		*P-*values

		Advanced Level Response n (%), N = 129,695	Basic Level Response n (%), N = 18,126	
Age group, years				0.55
<1	7,618	6,670 (88)	948 (12)	
1–4	51,676	45,303 (88)	6,373 (12)	
5–9	20,992	18,367 (87)	2,625 (13)	
10–14	26,885	23,624 (88)	3,261 (12)	
15–18	40,650	35,731 (88)	4,919 (12)	
Gender				0.66
Female	68,822	60,518 (88)	8,304 (12)	
Male	77,714	68,278 (88)	9,436 (12)	
Missing	1,285	899 (70)	386 (30)	
Region				<0.001
Northeast	15,986	11,784 (73)	4,238 (27)	
South	68,387	61,750 (90)	6,637 (10)	
Midwest	25,029	22,243 (89)	2,786 (11)	
West	38,326	33,920 (89)	4,406 (11)	
Missing	93	34 (37)	59 (63)	
Urbanicity				<0.001
Urban	123,909	109,516 (88)	14,393 (12)	
Suburban	7,813	6,921 (89)	892 (11)	
Rural	9,280	7,975 (86)	1,305 (14)	
Wilderness	1,942	1,499 (77)	443 (23)	
Missing	4,877	3,784 (78)	1,093 (22)	

**Table 2:** Regression model: independent associations with receipt of Advanced Life Support-level care.

	Adjusted Odds Ratio	95% Wald Confidence Limits		*P*-values
Age in years	1.002	1.000	1.005	0.07
Gender: female vs male	1.006	0.97	1.04	0.72
Urbanicity:				
Wilderness vs urban	0.44	0.39	0.49	<0.001
Rural vs urban	0.79	0.75	0.84	<0.001
Suburban vs urban	1.01	0.94	1.09	0.80

**Table 3:** Antiepileptic medication administration by patient demographics and geographic locations.

	All Patients N = 129,733	Antiepileptic Medication Administered No. (%) N = 11,698	Antiepileptic Medication Not Administered, No. (%) N = 118,035	*P-*values
Age group, years				<0.001
<1	6,893	645 (9)	6,248 (91)	
1–4	49,992	4,064 (8)	45,928 (92)	
5–9	19,016	2,093 (11)	16,923 (89)	
10–14	22,566	1,869 (8)	20,697 (92)	
15–18	31,266	3,027 (10)	28,239 (90)	
Gender				<0.001
Female	58,960	5,879 (10)	53,081 (90)	
Male	70,218	5,793 (8)	64,425 (92)	
Missing	555	26 (5)	529 (95)	
Region				<0.001
Northeast	10,943	859 (8)	10,084 (92)	
South	61,752	5,948 (10)	55,804 (90)	
Midwest	20,889	1,914 (9)	18,975 (91)	
West	36,117	2,974 (8)	33,143 (92)	
Missing	32	3 (9)	29 (91)	
Urbanicity				0.66
Urban	110,977	10,005 (9)	100,972 (91)	
Suburban	6,347	589 (9)	5,758 (91)	
Rural	7,301	656 (9)	6,645 (91)	
Wilderness	1,408	116 (8)	1,292 (92)	
Missing	3,700	332 (9)	3,368 (91)	

**Table 4:** Regression model: independent associations with medication being administered.

Effect	Adjusted Odds Ratio	95% Wald Confidence Limits		*P-*values
Age (in years)	1.008	1.005	1.01	<0.001
Gender: female vs male	1.22	1.18	1.27	<0.001
Urbanicity: wilderness vs urban	0.99	0.91	1.08	0.27
Urbanicity: rural vs urban	1.01	0.93	1.11	0.78
Urbanicity: suburban vs urban	0.90	0.74	1.09	0.77

**Table 5:** Time from dispatch to medication administration (in minutes).

	N	Lower Quartile	Median	Upper Quartile	*P*-values
All patients*	11,509	11.77	16.75	24.00	
Age (years)					<.001
<1	631	11.42	15.82	23.43	
1–4	3,997	11.05	15.67	22.12	
5–9	2,047	11.82	17.00	23.58	
10–14	1,837	12.67	17.88	26.03	
15–18	2,997	12.08	18.00	25.22	
Gender					0.82
Female	5,879	11.72	16.72	24.00	
Male	5,793	11.85	16.80	24.00	
Missing	26	12.00	15.32	24.43	
Region					<.001
Northeast	842	12.00	18.00	25.17	
South	5,865	12.48	17.85	25.00	
Midwest	1,862	11.05	15.51	21.47	
West	2,937	10.67	15.33	22.53	
Missing	3	18.00	19.00	30.00	
Urbanicity					<.001
Urban	9,862	11.63	16.44	23.57	
Suburban	574	13.00	18.99	27.22	
Rural	638	12.03	17.43	26.00	
Wilderness	110	14.00	21.00	34.30	
Missing	325	12.57	18.55	25.00	

* Filtered to remove times <0 minutes and >180 minutes.

**Table 6:** Time from emergency medical services arrival on scene to medication administration (in minutes).

	N	Lower Quartile	Median	Upper Quartile	*P*-value
All patients*	11,382	5.43	9.63	16.00	
Age (years)					<.001
<1	626	5.00	9.00	15.00	
1–4	3,940	5.00	8.68	14.72	
5–9	2,023	5.58	9.88	16.00	
10–14	1,821	6.00	10.53	18.27	
15–18	2,972	5.91	10.31	17.50	
Gender					0.72
Female	5,734	5.40	9.45	16.10	
Male	5,623	5.45	9.87	16.00	
Missing	25	5.00	9.45	16.92	
Region					<.001
Northeast	836	5.91	10.83	17.00	
South	5,814	5.88	10.00	17.00	
Midwest	1,840	5.28	8.82	14.30	
West	2,889	5.00	8.88	15.58	
Missing	3	6.00	9.00	21.00	
Urbanicity					<.001
Urban	9,751	5.40	9.50	16.00	
Suburban	565	6.00	10.63	17.00	
Rural	638	5.17	9.84	16.00	
Wilderness	108	5.27	12.00	19.02	
Missing	320	5.83	10.21	16.93	

* Filtered to remove times <0 minutes and >180 minutes.

**Table 7:** Regression model: independent associations with timing to medication administration.

Effect	Estimated Mean Difference	Standard Error	*P*-values
Age (years)	0.16	0.02	<.001
Gender (male referent)	−0.03	0.23	0.14
Urbanicity:			
- Wilderness vs urban (referent)	6.84	1.17	<.001
- Rural vs urban (referent)	2.20	0.50	<.001
- Suburban vs urban (referent)	2.43	0.52	<.001
