# Supplementary material for: National Variation in EMS Response and Antiepileptic Medication Administration for Children with Seizures in the Prehospital Setting
Source: West J Emerg Med. 2023 Jul 17;24(4):805–13. doi: 10.5811/westjem.59396 (PMC10393459; doi:10.5811/westjem.59396)
Supplement: Supplementary file 1 [file wjem-24-805-s001.docx]

Supplemental Tables:

Supplement 1. Antiepileptic Medication Categorization

| Medication | Names used in NEMSIS (from eMedications.03) |
| --- | --- |
| Midazolam | Midazolam, Midazolam Hydrochloride, Versed, Midazolam 5MG/ML Injectable Solution, Midazolam Injectable Solution, Midazolam 1MG/ML, Midazolam 5 MG/ML |
| Lorazepam | Lorazepam, Ativan, Lorazepam 2MG, Lorazepam Injectable Solution, Lorazepam 1MG/ML, Lorazepam 2MG/ML Injectable Solution, Lorazepam 2MG/ML |
| Diazepam | Diazepam, Valium, Diastat, Diazepam Injectable Solution, Diazepam 5MG, Diazepam 10 MG |
| Other | Levetiracetam, Keppra |
|  | Fosphenytoin, Phenytoin |
|  | Phenobarbital, PHENobarbital 65 MG/ML |
|  | Clonazepam |

Supplement 2. Specific Medication Administered by Demographics

|  | All Patients, No.,  N=11,698 | Midazolam, No. (%)  N=9699 | Lorazepam, No. (%)  N=1368 | Diazepam, No. (%)  N=577 | Benzodiazepine (not otherwise specified),  No. (%)  N=39 | AED NOS, No. (%)  N=1 | Other,  No. (%)  N=14 |
| --- | --- | --- | --- | --- | --- | --- | --- |
| Age group, years  <1  5-9  10-14  15-18 | 645  4,064  2,093  1,869  3,027 | 534 (83)  3,422 (84)  1,730 (83)  1,553 (83)  2,460 (81) | 63 (10)  398 (10)  234 (11)  228 (12)  445 (15) | 45 (7)  227 (6)  125 (6)  76 (4)  104 (3) | 1 (0)  12 (0)  3 (0)  10 (1)  13 (0) | 0 (0)  1 (0)  0 (0)  0 (0)  0 (0) | 2 (0)  4 (0)  1 (0)  2 (0)  5 (0) |
| Sex  Female  Male  Missing | 5,879  5,793  26 | 4,908 (83)  4,772 (82)  19 (73) | 670 (11)  694 (12)  4 (15) | 270 (5)  306 (5)  1 (4) | 23 (0)  15 (0)  1 (4) | 0 (0)  0 (0)  1 (0) | 8  6  0 |
| Region  Northeast  South  Midwest  West  Missing | 859  5,948  1,914  2,974  3 | 621 (72)  4,865 (82)  1,522 (80)  2,688 (90)  3 (100) | 197 (23)  706 (12)  247 (13)  218 (7)  0 (0) | 38 (4)  342 (6)  137 (7)  60 (2)  0 (0) | 3 (0)  27 (0)  4 (0)  5 (0)  0 (0) | 0 (0)  0 (0)  0 (0)  1 (0)  0 (0) | 0 (0)  8 (0)  4 (0)  2 (0)  0 (0) |
| Urbanicity  Urban  Suburban  Rural  Wilderness  Missing | 10,005  589  656  116  332 | 8,577 (86)  407 (69)  411 (63)  58 (50)  246 (74) | 969 (10)  139 (24)  165 (25)  38 (33)  57 (17) | 421 (4)  39 (7)  72 (11)  18 (16)  27 (8) | 31 (0)  3 (1)  3 (0)  1 (1)  1 (0) | 1 (0)  0 (0)  0 (0)  0 (0)  0 (0) | 6 (0)  1 (0)  5 (1)  1 (1)  1 (0) |
